# Low calcium dialysate combined with CaCO_3_ in hyperphosphatemia in hemodialysis patients

**DOI:** 10.3892/etm.2013.1067

**Published:** 2013-04-17

**Authors:** ZHUO GAO, LI-DE LUN, XIN-LUN LI

**Affiliations:** Department of Nephrology, Air Force General Hospital, Beijing 100142, P.R. China

**Keywords:** low calcium dialysis, hyperphosphatemia, calcium-phosphate product, parathyroid hormone

## Abstract

This aim of this study was to observe the effects of the application of low calcium dialysate (LCD) combined with oral administration of CaCO_3_ in the treatment of hyperphosphatemia, as well as blood Ca^2+^, calcium-phosphate product (CPP), parathyroid hormone (PTH) and blood pressure in patients undergoing hemodialysis. Thirty-one maintenance hemodialysis (MHD) patients with hyperphosphatemia, but normal blood Ca^2+^, underwent dialysis with an initial dialy-sate Ca^2+^ concentration (DCa) of 1.50 mmol/l for six months and then with 1.25 mmol/l for six months. The patients who underwent dialysis with a DCa of 1.25 mmol/l were treated orally with 0.3 g CaCO_3_ tablets three times a day. In the third and sixth months [observation end point (OEP)] of the dialysis, the concentrations of Ca^2+^, phosphorus and intact PTH (iPTH) were measured; blood pressure and side-effects prior to and following dialysis were also observed. The Ca^2+^, CPP and iPTH levels increased (P<0.05) in the sixth month of treatment with a DCa of 1.50 mmol/l. However, the Ca^2+^ concentration declined to a certain degree, CPPs decreased significantly (P<0.05) and the iPTH concentration increased following treatment with a DCa of 1.25 mmol/l for six months. The incidence rate of adverse effects of LCD was 12.9% (4/31); the effects were mainly muscle spasms, hypotension and elevated PTH. The periodic application of LCD combined with the oral administration of CaCO_3_ effectively reduced serum phosphorus and CPPs among MHD patients with hyperphosphatemia, indicating that the treatment may be used clinically.

## Introduction

Previously, several studies have reported that a high concentration of Ca^2+^ in the blood, hyperphosphatemia and increased calcium-phosphate products (CPPs) are major risk factors for the occurrence of cardiovascular calcification in hemodialysis patients (HPs) (1–4). High-level hyperphosphatemia often occurs in HPs. Conventional hemodialysis has limited effects on the removal of phosphorus (700–800 mg/oz) (5). In addition, for a number of patients it is not possible to increase the frequency of dialysis or to use an expensive, non-calcium phosphate binder to lower the amount of phosphorus due to economic conditions. Thus, CaCO_3_ remains the most commonly used phosphate binder in clinics.

Given that CaCO_3_ is a calciferous phosphorus-binding agent, it is likely to cause severe hypercalcemia and increase CPPs if applied inappropriately (6). Therefore, we established an economical, safe and effective method for reducing the concentration of phosphorus in the blood. We consider that this method may be applied in the clinic.

Use of a low calcium dialysate (LCD) is a method for reducing the calcium load, which effectively reduces CPPs, as well as the incidence and extent of metastatic calcification in dialysis patients. Currently, it is not known whether the oral administration of CaCO_3_ at different intervals will cause the amount of phosphorus in the blood and CPPs to decrease. Additionally, the safety of long-term application of LCD, whether LCD leads to increased QTc dispersion in the electrocardiogram (ECG) of dialysis patients and whether it induces ventricular arrhythmias (7,8) remain unknown. Furthermore, it has not been determined whether fluctuations in parathyroid hormone (PTH) levels and unstable blood pressure occur.

Calcium and phosphorus metabolism (CPM) disorders are among the most common and significant complications in HPs, among which, hyperphosphatemia, elevated CPPs and abnormal intact PTH (iPTH) level are the important risk factors for cardiovascular disease (9,10). The maintenance of appropriate levels of Ca^2+^, phosphorus and iPTH has become the main focus. In developing countries, the use of expensive non-calcium phosphate binders is not generally possible (11); thus, patients take large doses of calcium-containing phosphorus binders (CCPB) to treat hyperphosphatemia, including CaCO_3_, which increases the risk of hypercalcemia. The application of LCD reduces the Ca^2+^ load and allows the use of CCPB. This study observed the effects of periodic LCD combined with oral administration of CaCO_3_ in the treatment of hyperphosphatemia, as well as on CPPs, iPTH levels and blood pressure in HPs, to establish a simple, practical, safe and effective method for reducing phosphorus levels.

The aim of the current study was to determine whether the oral administration of CaCO_3_ during the long-term application of LCD in maintenance hemodialysis (MHD) patients is an economical, effective and safe clinical treatment for reducing the blood phosphate concentration and correcting disorders of CPM.

## Subjects and methods

### Subjects

A total of 31 patients who underwent hemodialysis in the Blood Purification Center of Air Force General Hospital (Beijing, China) between January 2009 and December 2009 were selected for the present study. The group included 17 males and 14 females, with an average age of 45.9±8.7 years, who had been undergoing dialysis for >6 months. The cases with primary diseases included 12 cases of chronic glomerulonephritis, 2 cases of hypertensive renal damage, 11 cases of diabetic nephropathy, 2 cases of lupus nephritis, 2 cases of drug-induced renal damage and 2 cases with unknown causes. The patients did not experience primary disease activity for three months prior to the treatment and did not have acute infection, surgical trauma, severe heart failure, active liver disease, cancer or other complications. The biochemical indicators when the patients enrolled were adopted as the initial values. The selected subjects met the following requirements: Ca^2+^ >1 mmol/l, serum phosphorus >1.78 mmol/l and iPTH ≤300 pg/ml.

This study was conducted in accordance with the Declaration of Helsinki and with approval from the Ethics Committee of Air Force General Hospital. Written informed consent was obtained from all participants.

### Dialysis method

A dialysate with a dialysate Ca^2+^ concentration (DCa) of 1.5 mmol/l was applied for six months and the patients received CaCO_3_ tablets depending on the concentration of Ca^2+^ in the blood. A dialysate with a DCa of 1.25 mmol/l (the other components of the dialysate remained the same) was then utilized for another six months. During dialysis, 0.3 g CaCO_3_ tablets were administered three times a day. All patients underwent dialysis three times a week for 4.0–4.5 h each time. A Braun dialysis machine (B. Braun Melsungen AG Company, Tuttlingen, Germany) was used, with a blood flow of 180–300 ml/min and dialysate flow of 500 ml/min. The dialyzer used was a LOPS 15 (polysulfone membrane) dialyzer with a membrane area of 1.5 m^2^. The patients remained stable during the observation period. Patients were provided with continuous calcium and phosphorus dietary intake and the regular use of other medication was also maintained, including antihypertensive drugs, vitamins, iron and erythropoietin.

### Observation indicators

Patient age, gender, weight and other general information were recorded. Forearm venous blood was obtained before dialysis in the third and sixth months to determine the clinical biochemical indicators, including serum calcium, phosphorus, CPP and iPTH levels. Blood pressure and the occurrence of adverse reactions prior to and following hemodialysis were also observed. Serum Ca^2+^ (reference range, 1.9–2.45 mmol/l) and serum phosphorus (reference range, 0.9–1.34 mmol/l) were analyzed using an automatic biochemical analyzer; serum iPTH was measured by the radioimmunoassay method.

### Statistical analysis

Statistical analysis was conducted using SPSS 13.0 software (SPSS Inc., Chicago, IL, USA). The measured data are presented as mean ± standard deviation. Paired t-test was utilized for comparison of data. P<0.05 was considered to indicate a statistically significant difference.

## Results

### Changes in Ca^2+^ and phosphorus concentration

Among the 31 selected cases, 30 completed the observation process. A patient treated with LCD for three months aborted the treatment due to a rapid increase in iPTH levels to almost 450 pg/ml. A dialysate with a DCa of 1.50 mmol/l was employed for the dialysis. The concentrations of Ca^2+^ and phosphorus for the 30 patients who completed the observation were monitored at different periods.

The changes in the Ca^2+^ and phosphorus concentrations following each type of dialysis were observed. Table I shows that the serum level of Ca^2+^ after 1.25 DCa dialysis was significantly lower than that after 1.5 DCa dialysis, with no significant difference of phosphorus level between them.

The differences in the concentrations of Ca^2+^, phosphorus, CPP and iPTH in the third and sixth months of dialysis by the two methods (oral CaCO_3_ combined with LCD) were also observed. Table II shows that Ca^2+^, phosphorus, CPP and iPTH levels slightly increased following treatment with a DCa of 1.5 mmol/l, whereas the Ca^2+^ and phosphorus concentrations reduced significantly following treatment with a DCa of 1.25 mmol/l. The degree of reduction of the concentration of phosphorus was particularly evident. Although the Ca^2+^ levels declined, they remained within the normal range. CPPs were significantly reduced and iPTH levels significantly increased in the third month; they tended to stabilize in the sixth month (Table II).

### Blood pressure changes

The differences in blood pressure prior to and following a single session of dialysis by the two methods were monitored. Table III shows that, the contractive and diastolic pressure after 1.25 DCa dialysis were significantly lower than those after 1.5 DCa dialysis.

The differences in blood pressure before and after dialysis in the third and sixth months for the two methods (oral CaCO_3_ combined with LCD) were monitored. Table IV shows that no significant difference was observed in the blood pressure before and after 1.5 mmol/l DCa dialysis, whereas blood pressure decreased in all the cases, particularly for contractive blood pressure, when 1.25 mmol/l DCa dialysis was performed. However, the blood pressure levels remained within the normal range (Table IV).

### Side-effects

Four out of the 31 patients (12.9%) reported the occurrence of side-effects. Two cases exhibited muscle spasms, which normally occurred late in the dialysis. An intravenous bolus of calcium gluconate was administered when the spasms occurred to prevent remission. Following symptomatic treatment, the patients tolerated the adverse side-effects and finished the experiment. Hypotension was experienced by one patient. The symptoms of hypotension were relieved after modifying the dosage of the antihypertensive drugs. The patient then insisted on completing the observation. One patient also exhibited an excessive increase of iPTH following the application of LCD; the iPTH level reached 450 pg/ml. The patient aborted the treatment. LCD with a DCa of 1.50 mmol/l was applied and calcitriol was administered orally. No apparent arrhythmia was observed.

## Discussion

In this study, we aimed to establish an economical, safe and effective method for reducing serum phosphorus in HPs; therefore, the LCD method combined with oral administration of CaCO_3_ for MHD patients was proposed. We identified that the periodic application of LCD combined with orally administered CaCO_3_ for the treatment of hyperphosphatemia in HPs effectively reduced the levels of serum phosphorus and CPPs without apparent serious complications.

Within the six-month application of LCD, the levels of serum Ca^2+^ remained stable and did not appear abnormal, which may be attributed to the calcium phosphate binders (CaCO_3_) orally administrated to the patients.

Various LCD results have been reported by other studies (12–16). The main factors that affect the secretion of PTH are Ca^2+^, phosphorus and vitamin D levels. Hypocalcemia is the strongest stimulus of PTH secretion. Therefore, the imbalance in Ca^2+^ caused by the application of LCD may significantly stimulate the secretion of PTH (17). One study (18) confirmed that LCD alone significantly increases serum PTH, revealing that the increase of PTH is related to the decline of blood Ca^2+^ and that PTH is restored to the level in the previous dialysis prior to the next dialysis. Ferreira *et al* (19) performed a one-year study of hemodialysis patients and identified that the iPTH levels significantly increased. The present study also identified that the application of the 1.25 mmol/l DCa for three months increases the iPTH level significantly (P<0.05), which is in agreement with the changes in Ca^2+^ concentration. Lund *et al* (20) observed seven patients with secondary hyperparathyroidism and identified that a large dose of a calcium phosphate binder combined with LCD controls the amount of serum phosphorus and does not affect the iPTH level.

We performed a six-month study of LCD and identified that the serum PTH levels increased. Several patients did not tolerate long-term LCD treatment as their PTH level increased too rapidly; the PTH levels of the majority of the subjects increased only slightly and reached a stable level after 3–6 months, which is consistent with the results of related literature (21). The reason for this occurrence may be the oral administration of CaCO_3_ during LCD treatment, which allows the PTH level to remain stable.

Serum Ca^2+^ in the human body affects the blood pressure by changing the vascular tone and regulating cardiac contraction (22). A change in the serum Ca^2+^ level may also affect the blood pressure of HPs. Wang *et al* (23) identified that following LCD, the systolic, diastolic and mean blood pressure of HPs declined significantly. In this study, the systolic and diastolic blood pressure exhibited a downward trend after six months of LCD treatment, suggesting that LCD has a certain impact on blood pressure. This impact requires long-term observation of a larger number of cases. For HPs with hyperphosphatemia and high blood pressure that is difficult to control, periodic treatment with LCD is the better choice.

We identified that during treatment with LCD, certain side-effects also occurred, particularly muscle spasm, which became tolerable in the majority of cases following symptomatic treatment. Low blood pressure was also common; however, it was resolved by adjusting the dosage of the antihypertensive drugs. No side-effect related to arrhythmia was observed in the current study; however, ECG should be performed during LCD treatment for patients with an unstable cardiovascular system.

In conclusion, the periodic application of 1.25 mmol/l DCa dialysis combined with oral CaCO_3_ effectively reduced the level of serum phosphorus and CPPs, thereby reducing the risk of calcification of soft tissues. The proposed method is an economical, effective and feasible treatment for HPs with hyperphosphatemia. This approach minimizes the secretion of PTH as long as normal blood Ca^2+^ levels are maintained. The effect on blood pressure is resolved by adjusting the dosage of the antihypertensive drugs. However, long-term conditions of low calcium should be monitored since these conditions may lead to parathyroid cell proliferation and a continuous increase of PTH. Strict monitoring of serum Ca^2+^, phosphorus and iPTH should also be performed. The dialysate Ca^2+^ concentration should be adjusted when iPTH increases excessively. Moreover, ECG results should be monitored to avoid serious adverse reactions.

## Figures and Tables

**Table I:** Ca^2+^ and phosphorus concentrations before and after a single dialysis session of two types of dialysis (n=30).

Items	Ca^2+^ (mmol/l)	Phosphorus (mmol/l)
DCa of 1.5 mmol/l		
Pre-single-dialysis	2.38±0.37	2.54±0.51
Post-single-dialysis	2.72±0.42^a^	1.80±0.62^a^
DCa of 1.25 mmol/l		
Pre-single-dialysis	2.40±0.32	2.47±0.38
Post-single-dialysis	2.37±0.27^a,b^	1.77±0.59^a^

Data are presented as mean ± standard deviation.

a P<0.05, compared with pre-single-dialysis;

b P<0.05, compared with DCa 1.5 mmol/l dialysis. DCa, dialysate Ca^2+^ concentration.

**Table II:** Ca^2+^, phosphorus, CPP and iPTH levels at the initial stage and after 3 and 6 months of using two types of dialysis (n=30).

Items	Ca^2+^ (mmol/l)	Phosphorus (mmol/l)	CPP (mg^2^/dl^2^)	iPTH (pg/ml)
1.5 mmol/l DCa dialysis				
Initial stage of dialysis	2.38±0.37	2.54±0.51	63.96±13.71	97.89±69.44
Third month of dialysis	2.37±0.36	2.58±0.85	65.91±17.80	106. 83±71.23^a^
Sixth month of dialysis	2.47±0.22^a,b^	2.64±0.78^a,b^	69.73±14.43^a,b^	103.24±81.45^a,b^
1.25 mmol/l DCa dialysis				
Initial stage of dialysis	2.40±0.32	2.49±0.52	71.98±15.74	103.71±48.37
Third month of dialysis	2.34±0.42^a^	1.98±0.41^a,c^	50.85±17.67^a,c^	184.17±78.57^a,c^
Sixth month of dialysis	2.38±0.34^a–c^	1.93±0.36^a–c^	46.79±15.70^a–c^	168.92±93.83^a–c^

Data are presented as mean ± standard deviation. All indicators were tested in the blood drawn before the dialysis. P<0.05, compared with

a initial stage using the same dialysate;

b 3 months using the same dialysate;

c 1.5 mmol/l DCa dialysis. DCa, dialysate Ca^2+^ concentration.

**Table III:** Blood pressure before and after a single dialysis session of two types of dialysis (n=30).

Items	Contractive pressure (mmHg)	Diastolic pressure (mmHg)
1.5 mmol/l DCa dialysis		
Pre-single-dialysis	165.2±15.10	92.4±7.90
Post-single-dialysis	150.6±13.50	80.2±9.20
1.25 mmol/l DCa dialysis		
Pre-single-dialysis	168.4±15.00	90.2±8.10
Post-single-dialysis	138.2±12.60^a^	69.8±10.10^a^

Data are presented as mean ± standard deviation.

a P<0.05, compared with 1.5 mmol/l DCa dialysis. DCa, dialysate Ca^2+^ concentration.

**Table IV:** Blood pressure before and after dialysis in the third and sixth months of using two types of dialysis (n=30).

Items	Contractive pressure (mmHg)	Diastolic pressure (mmHg)
1.5 mmol/l DCa dialysis		
Pre-dialysis in the third month	150.2±7.60	82.6±7.30
Post-dialysis in the third month	158.0±9.20	88.1±6.70
Pre-dialysis in the sixth month	152.0±6.32	84.3±5.63
Post-dialysis in the sixth month	151.7±7.40	84.5±5.40
1.25 mmol/l DCa dialysis		
Pre-dialysis in the third month	151.20±17.14	72.23±12.88
Post-dialysis in the third month	143.39±13.45^b^	72.55±10.91^b^
Pre-dialysis in the sixth month	138.84±13.73^a,b^	71.11±9.74^b^
Post-dialysis in the sixth month	132.84±8.56^a,b^	67.86±8.84^a,b^

Data are presented as mean ± standard deviation.

a P<0.05, compared with the blood pressure in the third month;

b P<0.05, compared with 1.5 mmol/l DCa dialysis. DCa, dialysate Ca^2+^ concentration.
